# Retinal blood vessels extraction using probabilistic modelling

**DOI:** 10.1186/2047-2501-2-2

**Published:** 2014-01-27

**Authors:** Djibril Kaba, Chuang Wang, Yongmin Li, Ana Salazar-Gonzalez, Xiaohui Liu, Ahmed Serag

**Affiliations:** Department of Information Systems, Computing and Mathematics Brunel University, London, UK; Diagnostic Imaging and Radiology, Children’s National Medical Center, 24105 Washington, DC USA

**Keywords:** Retinal images, Vessel segmentation, Expectation maximisation

## Abstract

The analysis of retinal blood vessels plays an important role in detecting and treating retinal diseases. In this review, we present an automated method to segment blood vessels of fundus retinal image. The proposed method could be used to support a non-intrusive diagnosis in modern ophthalmology for early detection of retinal diseases, treatment evaluation or clinical study. This study combines the bias correction and an adaptive histogram equalisation to enhance the appearance of the blood vessels. Then the blood vessels are extracted using probabilistic modelling that is optimised by the expectation maximisation algorithm. The method is evaluated on fundus retinal images of STARE and DRIVE datasets. The experimental results are compared with some recently published methods of retinal blood vessels segmentation. The experimental results show that our method achieved the best overall performance and it is comparable to the performance of human experts.

## Introduction

Automated segmentation of retinal structures allows ophthalmologist and eye care specialists to perform large population vision screening exams for early detection of retinal diseases and treatment evaluation. This non-intrusive diagnosis in modern ophthalmology could prevent and reduce blindness and many cardiovascular diseases around the world. An accurate segmentation of retinal blood vessels (vessel diameter, colour and tortuosity) plays an important role in detecting and treating symptoms of both the retinal abnormalities and diseases that affect the blood circulation and the brain such as haemorrhages, vein occlusion, neo-vascularisation. However, the intensity inhomogeneity and the poor contrast of the retinal images cause a significant degradation to the performance of automated blood vessels segmentation techniques. The intensity inhomogeneity of the fundus retinal image is generally attributed to the acquisition of the image under different conditions of illumination.

Previous methods of blood vessels segmentation can be classified into two categories: (1) pixels processing based methods, and (2) tracking-based or vectorial tracking or tracing methods [1].

Pixel processing based methods use filtering and morphological pre-processing techniques to enhance the appearance of the blood vessels in the image. The enhanced image is then processed using techniques such as thinning or branching to classify pixel as either belonging to vessels or background. Hoover et al. [2] proposed a framework to extract blood vessel from retinal images using a set of twelve directional kernels to enhance the vessels before applying threshold-probing technique for segmentation. Mendoca et al. [3] presented a method to extract a vessel centreline then filled it using the global intensity characteristics of the image and the local vessel width information. Maritiner-perez et al. proposed a new segmentation method of blood vessels from red-free and fluorescein retinal images. This method is based on multiscale feature extraction, which used the first and second spatial derivatives of the image intensity that provides information about vessel topology. A multiple pass region growing procedure is applied to segment the vessels using both vessels feature information and spatial information. Zhang et al. [4] extracted the blood vessel tree by matched filter with first-order derivative of Gaussian. The method detects the blood vessels by thresholding the retinal image’s response to the matched filter, while the threshold is adjusted by the response of the image to the first-order derivative of Gaussian. Cinsdikici et al. proposed a method of retinal blood vessel detection using a combination of a hybrid model of matched filter and ant colony algorithm.

The drawback of these techniques is that some proprieties of blood vessels can only be applied in the segmentation process after a low level preprocessing and they generally output poor segmentation results on unhealthy retinal images that have the presence of lesions. They also need more computational power when the size of an image increases, thus a special hardware is required for real time processing.

The tracking based approaches or vectorial tracking or tracing included semi automated tracing and automated tracing. In the semi automated tracing methods, the user manually selects the initial vessel seed point. The methods are generally used in quantitative coronary angiography analysis. In fully automated tracing, the algorithms select automatically the initial vessel points and most of them use Gaussian functions to characterise a vessel profile model, which locates a vessel points for the vessel tracing. The tracking based methods are seen as single pass operations, which perform the vessels’ structure detection and recognition simultaneously. The advances of using these techniques are that, they are computational efficient and much faster than pixels processing methods because the algorithms avoid the processing of every pixels in the image and use only the pixels in the neighbourhood of the vessels structure.

Xu et al. [5] combined the adaptive local thresholding method and the tracking growth technique to segment retinal blood vessels. Staal et al. [6] proposed a retinal blood vessel segmentation in two dimensional colour retinal images. The method extracts the images ridges, which are used to compose primitives in the form of line elements. The line elements are used to partition an image into patches, then a feature vectors are computed for every pixel and classified a kappa NN-classifier and sequential forward feature selection. Chaudhuri et al. [7] presented a framework to segment the blood vessels in the retina. The method is based on the optical and spatial properties of the vessels, where the gray-level profile of the cross section of a blood vessel is approximated by a Gaussian-shaped curve and a matched filter is used to define the piecewise linear segments of blood vessels. Finally twelve different templates are designed to search for vessel segments along all possible directions. Salazar et al. [8] used an adaptive histogram equalisation and the distance transform algorithm to enhance the vessels appearance, then applied the graph cut technique to segment vessels. In [9] Yin et al. proposed an automated tracking approach to segment blood vessels in retinal images. The technique detects vessel edge points iteratively using a Bayesian model based on statistical sampling approach and the intensity profile of the vessel are approximated by a Gaussian model. New vessel edge points are defined based on local grey level statistics and expected vessel features.

The limitations of the tracking based approaches are that the multiple branches models are not always applicable and they do not perform well on disease retinal. The semi automated tracking methods also require manual input, which required more times. Thus they are not suitable for real time retinal image analysis.

## Methods

In this paper, we present a new automated method to extract blood vessels in retinal fundus images. The proposed method is divided into two main stages: the pre-processing and the probabilistic modelling. Our approach takes as first step the correction of the intensity inhomogeneity of the retinal image using a bias correction algorithm[10], then the appearance of blood vessels are enhanced with an adaptive histogram equalisation. A probabilistic model optimised by an expectation maximisation (EM) algorithm is used to extract the vascular tree from the processed images. Finally, a length filter is applied on the output of the EM algorithm to eliminate all the non-vessels pixels.

The experimental results are performed on two publicly available datasets. The STARE (STructured Analysis of the Retina) [2] images were provided by the Shiley Eye Center at the University of California, San Diego, and by the Veterans Administration Medical Centre in San Diego. The images of the DRIVE [6] were obtained from a diabetic retinopathy-screening program in The Netherlands and the screening population consisted of 400 diabetic subjects between 25-90 years of age.

### Bias correction

One of the major issues associated with fundus retinal images is the intensity inhomogeneity across the images, which causes a significant degradation to the performance of automated blood vessels segmentation techniques. The intensity inhomogeneity of the fundus retinal image is generally attributed to the acquisition of the image under different conditions of illumination. In order to overcome such a problem, we use the N4 algorithm of bias correction presented in [10] which is a modified version of the original bias correction proposed N3 algorithm [11] that includes a modified iterative update within a multi-resolution framework. If we consider a noise free retinal fundus image *v*(*x*), defined as
1

where *v*^′^(*x*) is the uncorrupted image, *f*(*x*) is the bias field and ,  and . The following iteration solution derived in [11] is used to define the uncorrupted image at the *n*^*th*^ iteration as
23

where  is the estimated residual bias field at the *n*^*th*^ iteration and  is the expected value of the true image given the current estimate of the corrected image and it is defined in [11]. *S*^∗^{.} is referred as the B-spline approximator or the smoothing operator. The iterative solution used to perform the bias correction is given by (3), where  and the initial bias field estimate  is equal to zero. The first iteration yields
4

For the second iteration, the iteration scheme uses the corrected log  to re-estimate the expected value of the true image , and the bias field estimate  is calculated by inspecting (4). The iteration solution is designed to converge such that the value of . Using (4) the total bias field estimate is obtained as
5

Thus, the total bias field estimate at the *n*^*t*^*h* iteration is derived as
6

**Figure 1:**
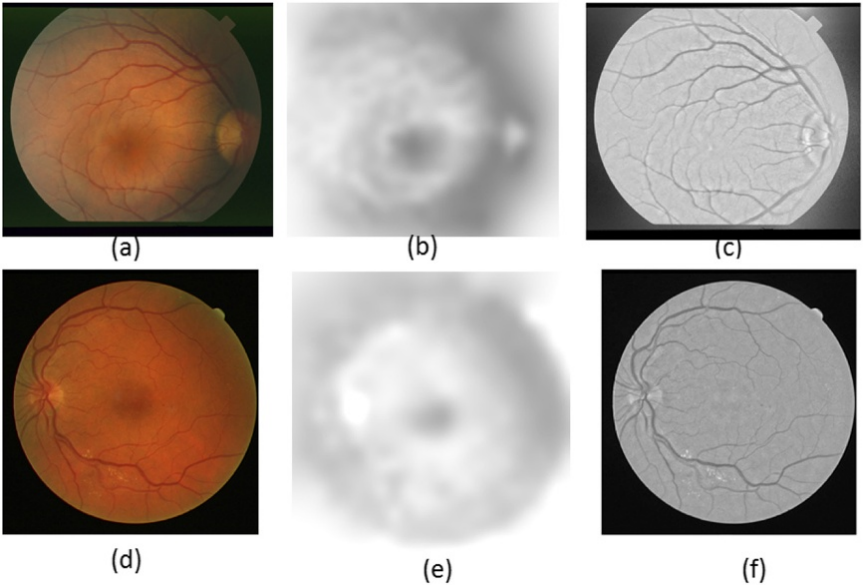
**Bias correction results.** **(a)** STARE image with intensity inhomogeneity. **(b)** Bias field. **(c)** Bias corrected image. **(d)** DRIVE image with intensity inhomogeneity. **(e)** Bias field. **(f)** Bias corrected image.

Figure 1 presents sample results of the bias corrected images.

### Adaptive histogram equalisation and distance transform

We apply an adaptive histogram equalisation [8] to the bias corrected image to enhance the contrast between vessel pixels and the background images. The histogram equalisation is performed using the flowing equation:
7

where


**Figure 2:**
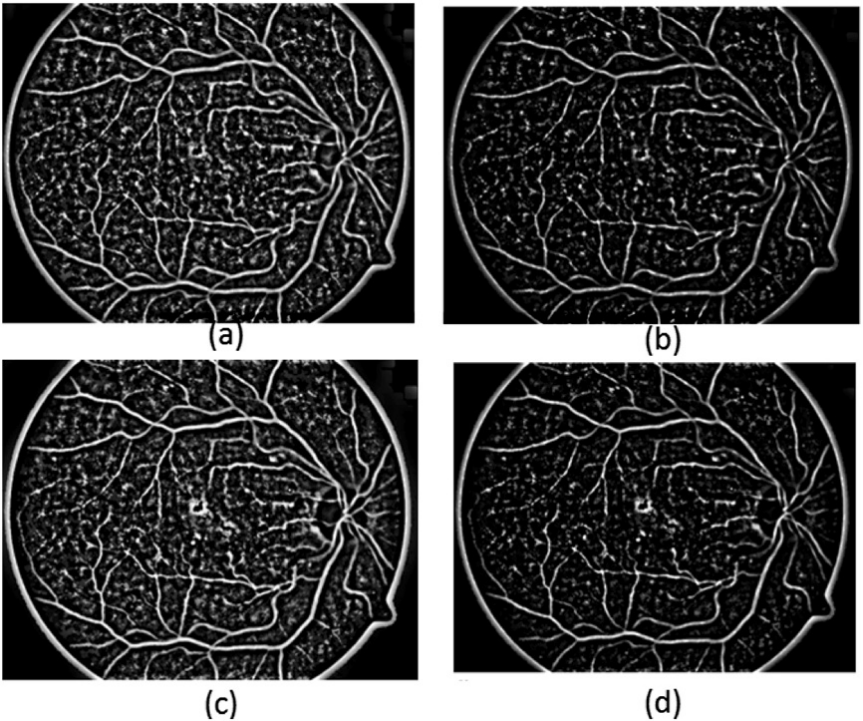
**Adaptive histogram equalisation results.** **(a)** *r*=3, *h*=45. **(b)** *r*=6, 45. **(c)** *r*=3, *h*=81. **(d)** *r*=6, *h*=81.

The notation *q* represents the pixels in the image and *q*^′^ is the neighbourhood pixels of *q*, defined by a square window of width *h*.The value of *r* indicates the level of contrast between the vessels and the background, by increasing the value of *r*, the contrast between vessel pixels and the background increases. Figure 2 shows the output images of the adaptive histogram equalisation with different values of *r* and *h*.

**Figure 3:**
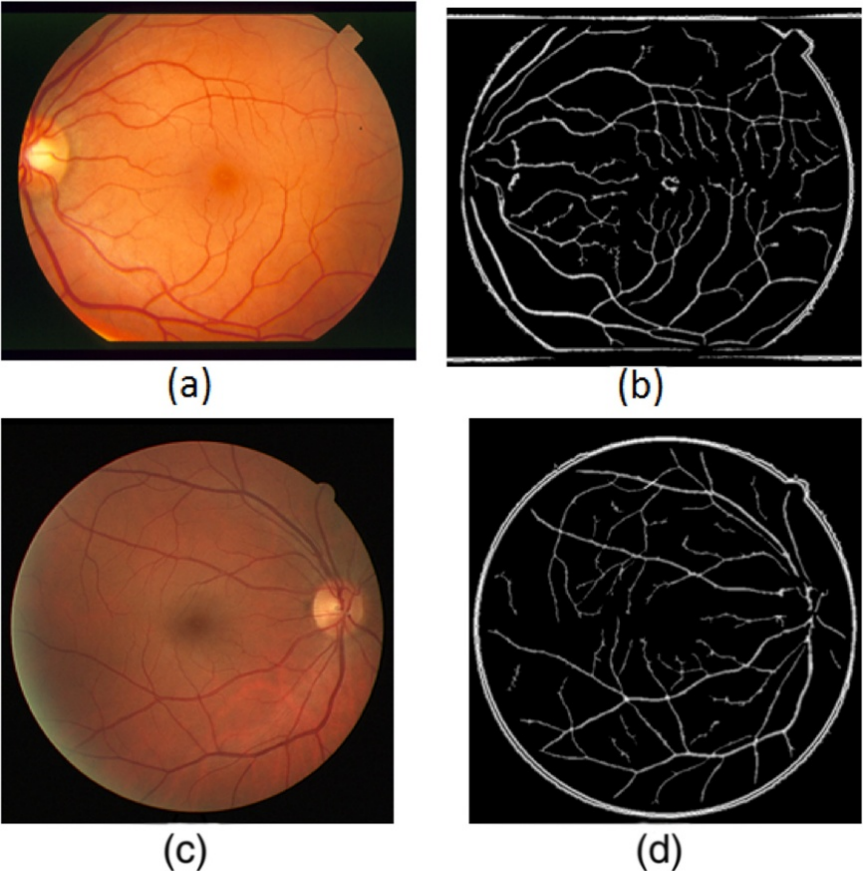
**Distance map images.** **(a)** STARE image. **(b)** STARE distance map. **(c)** DRIVE image. **(d)** DRIVE distance map.

To reduce the noise in the adaptive histogram equalisation image, a binary morphological open process is used to prune the image by eliminating all the non-vessels pixels. The pruned image is used to create a distance map image using a distance transform model. Finally, a probabilistic modelling is applied to the distance map image to extract the vessel tree. Figure 3 shows different fundus retinal image datasets with their corresponding distance map images.

### Probabilistic modelling

The extraction of the blood vessels is modelled with probabilistic unobserved variable model. An unobserved variable is introduced to model the process that determines the component from which the pixels observation originates. We introduce a binary vector  having a 1−*of*−*K* representation in which only one of the two elements in *U*_*k*_ can be equal to 1 and all other elements are equal to 0. *U*_*k*1_=1 if the *i*^*th*^ pixels in the retinal image  (where *M* is the number of pixels) can be accurately assigned to *K* clusters as vessel’s pixel otherwise *U*_*k*0_=1. A marginal distribution or a prior probability over *U*_*k*_ is defined such that *P*(*U*_*k*_=1)=*π*_*k*_ where *k*=1,…*K* thus:
8

where the probability values {*π*_*k*_} must satisfy 0≤*π*_*k*_≤1 and . The aim in this process is to estimate the unknown parameters representing the mixing value between the Gaussians and the means (*μ*_*k*_) and covariances (*Σ*_*k*_) of each component *θ*_*k*_=(*μ*_*k*_,*Σ*_*k*_). Thus, the conditional distribution or posterior probability of *X*_*i*_ given a particular value for *U*_*k*_ is defined as a mixture of two Gaussian distribution.
9

The joint probability is derived as the product of equations (8) and (9) to give
10

where the joint probability *P*(*X*_*i*_,*U*_*k*_∣*θ*_*k*_) defines a mixture of Gaussian mixture, and this model structure has been used in many problems of classification such as [12]. Assuming that  are independent and identically distributed and  is an unobserved variable, the likelihood is derived by marginalising *P*(*X*_*i*_,*U*_*k*_∣*θ*_*k*_) over the unobserved variable. In other word, the marginalise distribution of  is derived by adding the joint distribution over all possible states of . Our aim is to maximise the likelihood function that is given by
11

where *P*(*X*) is also a Gaussian mixture as the joint probability *P*(*X*_*i*_,*U*_*k*_∣*θ*_*k*_). As in vector *U*_*k*_ only one element can be equal to 1, the multiplication and summation over *k* in equation (11) can be the exhaustive summation of all possible values of  over *k*. Thus
12

### Expectation maximisation

To calculate the maximum likelihood estimate of the equation (11), we use the expectation maximisation (EM) algorithm as it is the most powerful method for finding maximum likelihood solutions for models with latent variables [13]. The EM performs the segmentation by classifying vessel’s pixels in one class (foreground) and non-vessel’s pixels in the other (background). The EM output is obtained by iteratively performing two steps: the expectation E- step computes the expected value of the likelihood function (pixel class membership function) with respect to the unobserved variables, under the parameters of a Gaussian mixture model and the maximisation M-step, maximises the likelihood function defined in the E-step until convergence [14]. In the E-step, the posterior probability *P*=(*U*_*k*_∣*X*_*i*_,*θ*_*k*_) of the unobserved variable *U*_*k*_ is derived using using Bayes’ theorem as:
13

Therefore the expectation of the unobserved variable *U*_*k*_ is derived with respect to the distribution of the posterior probability or the responsibility *Γ*(*U*_*k*_) that component *k* takes for assigning pixel *X*_*i*_ in the E-step. Then follow by the M-step, which calculates parameters maximising the expected log likelihood computed in the E-step. Suppose that the number of pixels in a retinal image is represented by a data set {*x*_*i*_,…*x*_*M*_} and we aim to model this set using a mixture of Gaussians. An *M*×*D* matrix *X* is used to represent the pixel data set in which the *i*^*th*^ row is defined by . The corresponding latent variables is represented by a matrix *Z* of size *M*×*K* in which the *i*^*th*^ row is defined by . Assuming that the pixel data points are drawn independently from the Gaussian distribution, we can define the log of the likelihood function using equation (12)
14

The derivative of ln{*P*(*X*∣*θ*)} with respect to the means *μ*_*k*_ of the Gaussian components is set to 0 to give.
15

By multiplying (15) by , we define the means as
16

where  is the total number of pixels assigned to cluster *k*. We can observed from equation (16) that the mean for the *k*^*th*^ Gaussian component *μ*_*k*_ is defined by using a weighted mean of all of the pixels in the data set, where the weighting factor for the image pixel point *x*_*i*_ is derived using the posterior probability *Γ*(*u*_*ik*_). Therefore a Gaussian component {*k*} is responsible for generating the image pixel points *x*_*i*_.

Similarly, we maximise ln{*P*(*X*∣*θ*)} with respect to the covariances *Σ*_*k*_ and we obtain
17

Like the mean *μ*_*k*_, the denominator of (17) is defined by the total number of pixel points assigned to cluster *k* and each pixel point is weighted by the corresponding posterior probability.

Finally, setting the derivative of ln{*P*(*X*∣*θ*)} with respect to (*π*_*k*_) the mixing coefficients and by using a Lagrange multiplier to satisfy the constraint , we obtain
18

By multiplying both sides of equation (18) and summing over *k*, we obtain the mixing coefficient as
19

From (19), we can see that the expression of the mixing coefficient for a component *k* is defined by the average responsibility that component *k* for assigning image pixels.

**Figure 4:**
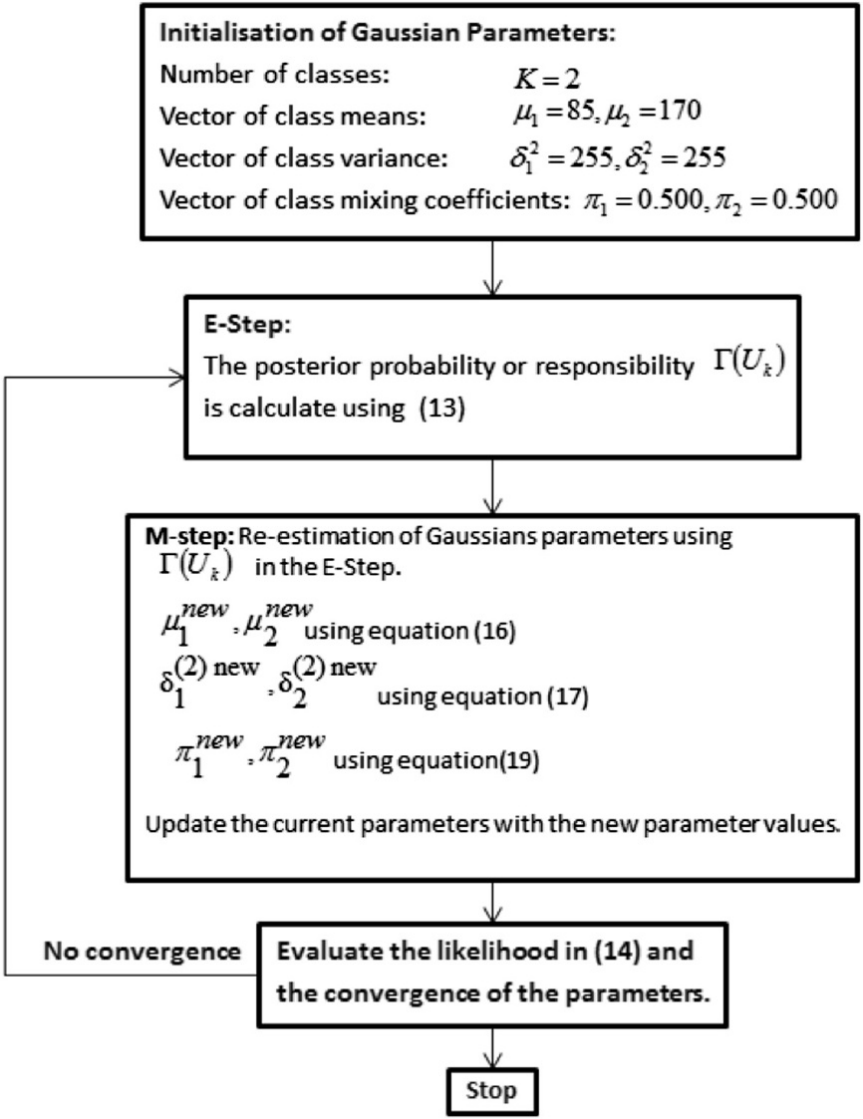
**The EM algorithm summary.** The steps of EM algorithm.

**Figure 5:**
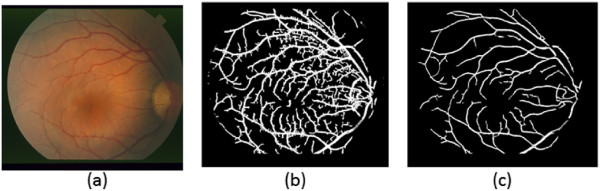
**The EM algorithm and length filter results.** **(a)** Fundus retinal image. **(b)** The EM algorithm output image. **(c)** the Length filter output image.

In all, to perform the EM algorithm, we first choose initial values for Gaussians parameters (means, covariance and mixing coefficients), then the algorithm iterates between the E-step and the M-step [14]. The EM algorithm process is summarised in Figure 4. In the E-step, the currents values of the parameters are used to calculate the values of the *Γ*(*u*_*ik*_) the posterior probabilities (responsibilities) given by equation (15). These probabilities values are used in the M-step to re-calculate the values of the Gaussians parameters means, covariance and mixing coefficients derived in (16), (17), (19) respectively. However each update to the Gaussians parameters from the E-step and the M-step is guaranteed to increase the log likelihood. Figure 5 shows the experimental results of the EM algorithm and the Length filter, where (a) is the input retinal image, (b) is the output of the EM algorithm and (c) the Length filter result.

### Length filter

In Figure 5(b), the result of the EM algorithm shows some misclassified pixels which increases the false positive. To address this problem, the length-filtering model is designed to eliminate all the non-vessels pixels in the EM algorithm result image. We adapt the length filtering used in [15], which discard all the groups of pixel with pixel number less than a certain number of pixels. The approach uses connected pixels labelling model, in which each individual object in the image is defined as connect regions. The approach starts by identifying all the connected regions, then discard all the connected objects less than a certain number of pixels using an eight-connected neighbourhood of all surrounding pixels. Finally label propagation is used and all connected components larger than a certain number of pixels are labeled as blood vessels. This approach reduces significantly the false positive, the output of the length filtering is shown in Figure 5(c).

## Experimental results

The method presented in this paper was evaluated on two publicly available retinal image datasets: STARE presented by Hoover et al. [2] and the DRIVE by Staal et al. [6]. The STARE dataset contains 20 fundus colour retinal images, including 10 healthy and 10 unhealthy ocular fundus images. The images are captured by a Topcon TRV-50 fundus camera at 35° field of view (FOV) and the size of the image is 700×605 pixels. The dataset provides two sets of hand labelled images segmented by two human experts as ground truth for retinal vessel segmentation methods. We calculated the mask of the image for this dataset using a simple threshold technique for each colour channel. We adapt the first expert hand labelled as the ground truth to evaluate our segmentation technique.

The DRIVE dataset provided 40 fundus colour ocular images, including 20 training images and 20 test images. These images are acquired using The Canon CR5 camera at 45° FOV, digitised at 24 bit with resolution of 565×584 pixels. The dataset also provides two sets of hand labelled images segmented by two human experts as ground truth. The first expert hand labelled was adapted as ground truth in the evaluation on both the STARE and the DRIVE datasets.

To facilitate the performance comparison between our method and other retinal blood vessels segmentation methods, the parameters measuring the performance (true positive rate, false positive rate and the accuracy rate) of [6], [2], [3] were used to measure the performance of the segmentation. The true positive rate (TPR) is defined as the ratio of the total number of pixels correctly classified as vessel pixels to the total number of vessel pixels in the image ground truth. The false positive rate (FPR) is the ratio of the total number of non vessel pixels in the FOV classified as vessel pixels to the total number of non vessel pixels inside the FOV of the the ground truth image. Finally the accuracy (ACC) is computed as the sum of true positives and true negatives over the total number of pixels in an given image. It is worth mentioning that a perfect segmentation would have a FPR of 0 and a TPR of 1. All the methods used the first expert hand labelled images as performance reference.

### STARE dataset

The experiment results of different retinal blood vessels segmentation methods on the STARE dataset are shown in Tables 1 and 2. The performance results of Staal et al. [6], Mendonça et al. [3], Martinez-Perez et al. [16], Chaudhuri et al. [7], Zhang et al. [4], and Hoover et al. [2] were generate from their original manuscripts. The performance of the different methods was generated using all the 20 fundus images except the method presented Staal [6], that used 19 out of the 20 images including 10 healthy and 9 unhealthy images. Our method has the highest TPR and with an average accuracy of 0.9456, it performs better than the methods presented by Mendoca et al. [3], Hoover et al. [2], Chaudhuri et al. [7] and Maritiner-Perez et al. [16] and its only marginally inferior to the method presented by Staal et al. [6] and hang et al. [4]. However as mentioned above the method presented by Staal et al. uses only 19 images for performance evaluation.

**Table 1:** **The performance comparisons on STARE dataset (Healthy and unhealthy retinal images)**

Method	TPR	FPR	Accuracy
2^*nd*^ human observer [3]	0.8949	0.0610	0.9354
Mendonca [3]	0.6996	0.0270	0.9440
Staal [6]	0.6970	0.0190	0.9516
Chaudhuri [7]	0.6134	0.0245	0.9384
Maritiner-Perez [16]	0.7506	0.0431	0.9410
Hoover [2]	0.6751	0.0433	0.9267
Zhang [4]	0.7177	0.027	0.9484
Our segmentation method	**0.7619**	**0.0328**	**0.9456**

**Table 2:** **The performance comparisons on STARE dataset (Healthy vs Unhealthy retinal images)**

Method	TPR	FPR	Accuracy
**Unhealthy ocular images**			
2^*nd*^ human observer [3]	0.8252	0.0456	0.9425
Mendonca [3]	0.6733	0.0331	0.9388
Hoover [2]	0.6736	0.0528	0.9211
Chaudhuri [7]	0.5881	0.0384	0.9276
Zhang [4]	0.7166	0.0327	0.9439
Our segmentation method	**0.7068**	**0.0324**	**0.9417**
**Healthy ocular images**			
2^*nd*^ human observer [3]	0.9646	0.0764	0.9283
Mendonca [3]	0.7258	0.0209	0.9492
Hoover [2]	0.6766	0.0338	0.9324
Chaudhuri [7]	0.7335	0.0218	0.9486
Zhang [4]	0.7526	0.0221	0.9510
Our segmentation method	**0.8506**	**0.0300**	**0.9554**

We also compared the performance of our method on both healthy and unhealthy ocular images. The results of the experiments show that the unhealthy ocular images cause a significant degradation to the performance of automated blood vessels segmentation techniques. Table 2 shows that on both images, our method outperforms the methods proposed by Mendoca et al. [3], Chaudhuri et al. [7] and Hoover et al. [2] and it is comparable to the performance of human experts.

### DRIVE dataset

Similarly to STARE dataset, The performance results of Staal [6], Mendonça [3], Martinez-Perez [16], Chaudhuri [7], Perfetti [17], Garq [18], Al-Rawi [19], Cinsdikici [20], Marin [21] and Zhang [4] were generated from their original manuscripts. But the performance results of Zana [22] and Jiang [23] techniques were provided by Staal [6] as their manuscripts were published before the DRIVE dataset was available. The performance of all the methods was measure on the 20 test images and the results are shown in Table 3. An overview of the testing results show that our method outperforms all other methods in term of TFR and with the accuracy, its marginally inferior to the method presented by Staal et al. [6], Marin et al. [21], Mendonça et al. [3] and the performance of human experts. Nevertheless it is important to note that the methods presented Staal et al. and Marin et al. used supervised techniques that generally depend on the training datasets, hence good segmentation results are achieved by classifier retraining before experimentations on new datasets.

**Table 3:** **The performance comparisons on DRIVE dataset**

Method	TPR	FPR	Accuracy
2^*nd*^ human observer [4]	0.7761	0.0275	0.9473
Mendonca [3]	0.7344	0.0236	0.9452
Staal [6]	0.7194	0.0227	0.9442
Chaudhuri [7]	0.6168	0.0259	0.9284
Maritiner-Perez [16]	0.7246	0.0345	0.9344
Jiang [23]	-	-	0.9112
Perfetti [17]	-	-	0.9261
Zana [22]	-	-	0.9377
Garq [18]	-	-	0.9361
Marin [21]	-	-	0.9452
Al-Rawi [19]	-	-	0.9510
Cinsdikici [20]	-	-	0.9293
Zhang [4]	0.7120	0.0276	0.9382
Our segmentation method	**0.7466**	**0.0317**	**0.9410**

**Figure 6:**
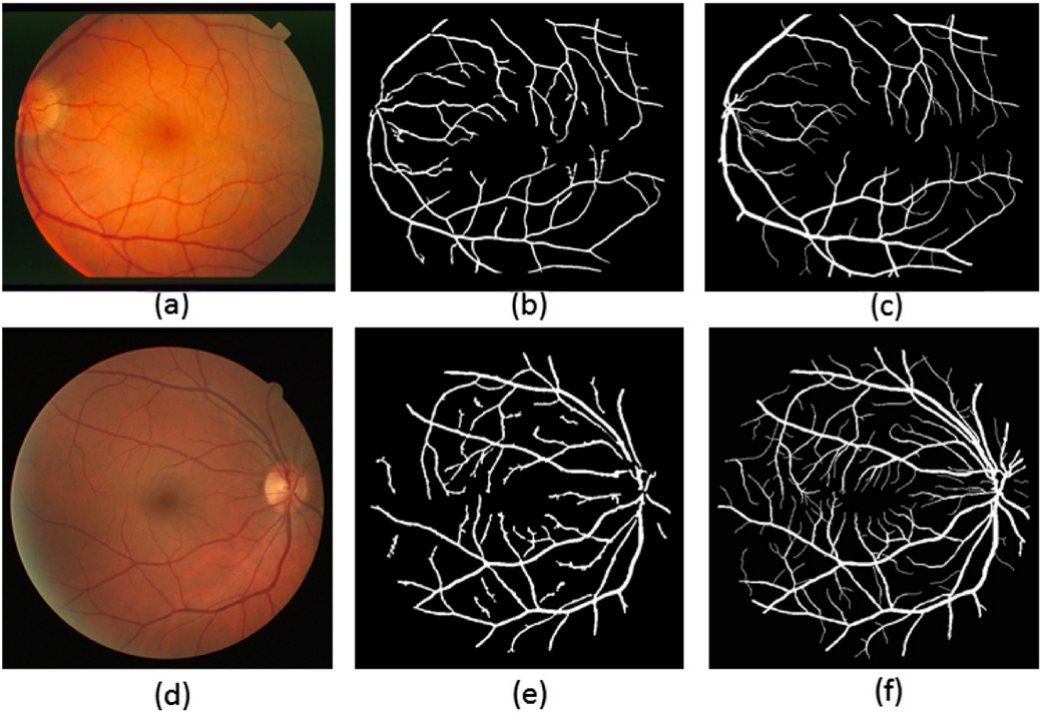
**The sample results of our method.** **(a)** STRARE fundus image. **(b)** Our method result. **(c)** Ground truth. **(d)** DRIVE fundus image. **(e)** Our method result. **(f)** Ground truth.

Figure 6 shows our experimental results where (a and d) are the input fundus retinal images, (b and e) the result of our method and (c and f) the ground truth. The images are from STARE and DRIVE respectively.

## Conclusions and discussion

We have presented in this paper a new approach to blood vessels segmentation by integrating the pre-processing techniques such bias correction and distance transform with a probabilistic modelling EM segmentation method. We have evaluated our method against other retinal blood vessels segmentation methods on STARE and DRIVE datasets. The overview of the experimental results presented in Tables 1, 2 and 3 show that the proposed approach achieved the best overall performance.

Our method has an advantage over tracking-based methods because it applies bias correction and distance transform on retinal images to enhance vessel appearance and allows multiple branches models. Also our method achieves better results over pixel processing based methods as it corrects the intensity inhomogeneities across retinal images to improve the segmentation of the blood vessels. This technique also minimises the segmentation of the optic disc boundary and the lesions in the unhealthy retinal images.

## Authors’ information

Djibril Kaba is the first author and has made more contributions, the rest of the authors have made the same contributions.
